# Safety and pharmacokinetics of BI 685509, a soluble guanylyl cyclase activator, in patients with cirrhosis: A randomized Phase Ib study

**DOI:** 10.1097/HC9.0000000000000276

**Published:** 2023-10-27

**Authors:** Eric J. Lawitz, Thomas Reiberger, Jörn M. Schattenberg, Corinna Schoelch, Harvey O. Coxson, Diane Wong, Judith Ertle

**Affiliations:** 1 The Texas Liver Institute, University of Texas Health, San Antonio, Texas, USA; 2 Division of Gastroenterology and Hepatology, Department of Medicine III, Medical University of Vienna, Vienna, Austria; 3 Christian Doppler Laboratory for Portal Hypertension and Liver Fibrosis, Medical University of Vienna, Vienna, Austria; 4 Metabolic Liver Research Program, I. Department of Medicine, University Medical Center Mainz, Mainz, Rhineland Palatinate, Germany; 5 Boehringer Ingelheim Pharma GmbH & Co. KG, Biberach, Germany; 6 Translational Medicine and Clinical Pharmacology, Boehringer Ingelheim Pharmaceuticals, Inc., Ridgefield, Connecticut, USA; 7 Boehringer Ingelheim International GmbH, Ingelheim am Rhein, Germany

## Abstract

**Background:**

Portal hypertension is a severe complication of cirrhosis. This Phase Ib study (NCT03842761) assessed the safety, tolerability, and pharmacokinetics of soluble guanylyl cyclase activator BI 685509 in patients with mild or moderate hepatic impairment (Child–Pugh [CP] A or B cirrhosis) and healthy volunteers (HVs).

**Methods:**

In this single-center, randomized, placebo-controlled study, patients received BI 685509 (maximum doses: 1, 2, or 3 mg, twice daily [BID]) or placebo for 28 days. HVs received one 0.5 mg dose of BI 685509 or placebo.

**Results:**

In total, 64 participants (CP-A, n=24; CP-B, n=25; HVs, n=15) were included; most commonly with NAFLD (36.7%), alcohol-associated (30.6%), or chronic viral hepatitis-related cirrhosis (28.6%). In patients with CP-A cirrhosis, drug-related adverse events (AEs) occurred in 5.6% of BI 685509-treated patients and 16.7% of placebo recipients. In patients with CP-B cirrhosis, drug-related AEs occurred in 26.3% of BI 685509-treated patients only. No serious AEs occurred in patients with CP-A cirrhosis; in patients with CP-B cirrhosis, serious AEs (not drug-related) occurred in 10.5% of BI 685509-treated patients and 16.7% of patients receiving placebo. BI 685509 was rapidly absorbed; exposure increased with dosage and was similar between etiologies and between patients with CP-A cirrhosis and patients with CP-A cirrhosis but lower in HVs. The mean percentage portal–systemic shunt fraction was measured in patients with CP-A cirrhosis and decreased at the end of treatment in the 2 mg BID (–11.2 ± 11.9%) and 3 mg BID (–14.0 ± 8.4%) BI 685509 dose groups, but not in the placebo group (+1.0 ± 27.3%).

**Conclusion:**

BI 685509 was generally well tolerated, with 3 serious, not drug-related AEs reported in patients with CP-B cirrhosis. In patients with CP-A cirrhosis, portal–systemic shunt fraction in the exploratory efficacy analysis was reduced by 2 mg BID and 3 mg BID BI 685509.

## Graphical abstract

**Figure:**
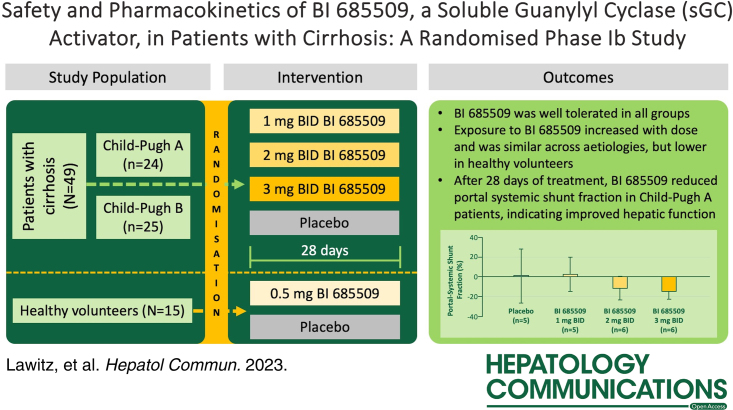

## INTRODUCTION

Cirrhosis is a devastating outcome of chronic inflammation in the liver, with over 122 million cases of cirrhosis resulting in 1.32 million deaths worldwide.^1^ Cirrhosis arises due to a wide array of factors and is associated with various complications, the most prevalent being portal hypertension (PH),^2^ which can ultimately lead to decompensation events (ascites, variceal hemorrhage, or HE) and mortality.^3–5^ While there are no treatments that specifically target PH,^2,6^ off-label nonselective beta-blockers (NSBBs) to reduce portal venous inflow^7^ and endoscopic variceal ligation to prevent bleeding are commonly used. However, many studies suggest that there is a narrow “therapeutic window” for the use of NSBBs between the development of esophageal varices and the complications of advanced cirrhosis.^8^ Moreover, considering that ligation does not influence the fundamental issue of PH^2,6^ and may potentially trigger severe ulcer bleeding associated with the procedure,^9^ this approach must be employed with caution and limited to select patients. Therefore, there remains a large unmet need for effective therapies that directly target PH.

Disrupted nitric oxide (NO) synthesis is a hallmark of PH pathophysiology.^10^ The primary receptor for NO is soluble guanylyl cyclase (sGC), which, upon activation by NO, catalyzes the formation of cyclic guanosine monophosphate from guanosine triphosphate,^11,12^ promoting vasodilation and smooth-muscle relaxation.^13^ Studies show that both NO and cyclic guanosine monophosphate production decrease in models of cirrhosis, leading to HSC activation.^14,15^ Activated HSCs constrict hepatic sinusoids by adopting contractile characteristics causing restricted portal flow, promoting venous congestion. In addition, they transdifferentiate into myofibroblasts and produce excess extracellular matrix proteins, resulting in fibrosis as the main structural component of increased hepatic vascular resistance.^16–18^

Recently developed sGC modulators exhibit diverse effects across cardiac, renal, and liver diseases, including reduction of fibrosis and portal pressure,^13,19^ impacting both dynamic (vasoactive) and static (fibrosis) PH components.^20,21^ Further studies have shown that sGC activators bind to the heme-binding domain, mimicking NO-bound heme and activating the NO-unresponsive form of sGC.^22^ This suggests that sGC activators have greater pharmacological activity than sGC stimulators, such as BAY 41-2272, under pathophysiological conditions and oxidative stress.^23^ Therefore, sGC activators represent a potential therapeutic avenue for conditions such as clinically significant PH (HVPG ≥10 mm Hg^24^).

BI 685509 is an NO-independent sGC activator currently being investigated in patients with PH to slow cirrhosis progression and reduce portal pressure. This Phase Ib clinical trial examined the safety, tolerability, and pharmacokinetics (PK) of BI 685509 in patients with mild (Child–Pugh [CP]-A) or moderate (CP-B) hepatic impairment, using different dosing schemes and compared single doses of BI 685509 in patients and individually matched healthy volunteers (HVs).

## METHODS

### Study design and participants

This single-center, randomized, placebo-controlled, double-blind (within dose groups), parallel-group, dose-escalation, Phase Ib study investigated 3 dosing schemes of BI 685509 in patients with mild or moderate hepatic impairment and compared single BI 685509 doses in patients with matched HVs. The planned enrolment was 64 participants: 48 patients with hepatic impairment (24 each per CP-A/CP-B), and 16 HVs.

All eligible participants were aged ≥18 years, with a MAP of ≥85 mm Hg and an estimated glomerular filtration rate of >70 mL/min/1.73 m^2^ at screening. Patients with hepatic impairment were classified into 2 groups by CP score (CP-A5/A6 for compensated cirrhosis and CP-B7/B8/B9 for decompensated cirrhosis), and all patients had liver stiffness > 15 kPa. Patients with CP-A cirrhosis did not show any clinical evidence of decompensation; however, previous decompensation events that were self-limited and resolved were allowed if they had occurred ≥ 6 weeks before screening and did not require continued therapeutic intervention at screening. HVs were included if they were deemed healthy by the investigator’s assessment, and they were individually matched to a patient with CP-A or CP-B (age ± 5 y, bodyweight ± 15%, sex).

Participants were excluded if they had ongoing chronic alcohol or drug use (which, in the investigator’s opinion, would have resulted in unreliable results or the inability to complete the trial), history of relevant orthostatic hypotension, major operation within 12 weeks prior to screening, primary sclerosing cholangitis, primary biliary cholangitis or HIV, or if they were pregnant or nursing. Patients with CP-B cirrhosis were excluded if they had refractory ascites or a recent decompensation event within 6 weeks of screening.

All patients provided written informed consent before study enrollment, and these studies were conducted in accordance with Good Clinical Practice (CPMP/International Council on Harmonisation/135/95), ethical principles laid down in the 2013 Declaration of Helsinki, and applicable regulatory requirements.

### Randomization and blinding

Participants within each patient group (CP-A/-B) were randomized 3:1 to receive oral tablets of BI 685509 (1 of 3 dose groups; maximum tested doses after dose escalation: 1 mg twice daily [BID], 2 mg BID, 3 mg BID) or placebo (Supplemental Table S1, http://links.lww.com/HC9/A535). A randomization list with study medication numbers, developed by the sponsor, was provided to the trial site. This randomization list was generated using a validated system involving a pseudo-random number generator. All trial participants, investigators, and site staff were blinded to the assigned treatment. Dose escalation was carried out sequentially, with a dose group receiving treatment only after the previous group had completed treatment and safety had been confirmed. HVs received a single dose of the same treatment as the individually matched patient (0.5 mg BI 685509 or placebo). Administered treatments were blinded within dose groups.

### Procedures

Treatment was administered to patients on site after an overnight fast on days 1, 4–5, 10–11, 16–17, and 27–28. Treatment administered at home (days 6–9, 12–15, and 17–26) was taken with a glass of water immediately before or during breakfast (and dinner if BID) at approximately the same time each day; for BID dosing, there was an interval of ≥ 10 hours between doses. On days 5, 11, and 17, the morning dose was administered on site, and the second dose was administered at home.

Assessments of PK, vital signs, BP, heart rate (HR), 12-lead ECG, orthostatic tests, safety laboratory tests, and CP status were carried out on site (days 1, 4, 10, 16, 28, and 31 for all; PK was assessed on days 2 and 3; PK and vital signs were assessed on days 27–31 inclusive). Liver stiffness (transient elastography) and fat content (controlled attenuation parameter) were assessed by FibroScan (any available model; Echosens, Paris, France) in a fasted state on days 1, 16, and 27. Spleen stiffness (transient elastography) was assessed by FibroScan Expert 630 Professional (Echosens) in compensated patients with CP-A cirrhosis on days 1, 16, 17, 27, and 28, also in a fasted state. Liver function was assessed by the HepQuant SHUNT test (HepQuant, Denver, CO, USA) in patients with CP-A cirrhosis at screening and on days 11 and 27, with 6 blood samples (each 3 mL) taken per test (ie, predose, and at 5, 20, 45, 60, and 90 min postdose). Adverse events (AEs) were assessed daily.

Treatment was administered to HVs on site after an overnight fast on day 1. PK were assessed on days 1–4; vital signs were assessed on days 1, 2, and 4; 12-lead ECG, physical examinations, and orthostatic tests were assessed on days 1 and 4; and AEs were assessed daily from screening to the end of observation (day 8).

### End points

The primary end point was the number of patients with drug-related AEs across different dose regimens over each dose escalation. Secondary safety end points were assessed after 28 days of treatment as the change from baseline in seated systolic BP, seated diastolic BP, HR, and bodyweight. Further safety end points were assessed based on treatment-emergent adverse events (TEAEs), safety laboratory parameters, vital signs, and 12-lead ECG.

Secondary PK end points were AUC from time 0 to the last quantifiable data point (AUC_0–tz_) and the observed C_max_ of BI 685509 after the first drug administration up to 72 hours in patients and HVs, and the AUC at steady state over a uniform dosing interval τ (AUC_τ,ss_) and C_max_ at steady state (C_max,ss_) of BI 685509 after the last drug administration up to 72 hours in patients with CP-A/B cirrhosis.

Exploratory efficacy end points in patients with CP-A and CP-B cirrhosis were the change from baseline in liver stiffness measured by FibroScan (any available model; Echosens) on days 16 and 27, and in patients with CP-A cirrhosis only, the change from baseline in spleen stiffness measured by FibroScan (Expert 630 Professional; Echosens) on days 16, 17, 27, and 28, and the change from baseline in liver function measured by the disease severity index, d4-cholate at 60 minutes (HepQuant STAT), and SHUNT using the HepQuant SHUNT test (HepQuant) on days 11 and 27. Additional exploratory end points included total serum levels of cytokeratin 18 (M65), caspase-cleaved cytokeratin 18 (M30), Pro-C3, alanine aminotransferase, aspartate aminotransferase, alkaline phosphatase, γ-glutamyltransferase, bilirubin and C-reactive protein, and composite scores (enhanced liver fibrosis test, NAFLD fibrosis, fibrosis-4, aspartate aminotransferase-to-platelet ratio index, and MELD. The changes from baseline after 27 days of treatment in patient-reported outcomes (EQ-5D-5L and chronic liver disease questionnaires) were defined as other end points.

### Statistical analyses

The planned sample size for this trial was not based on a power calculation. This study planned to include 64 participants: 16 HVs and 48 patients (8 patients per patient group [CP-A/-B] and dose group: 6 on active treatment, 2 on placebo; this is common in multiple rising dose studies of this type and is considered sufficient to detect major differences between dose groups and placebo in terms of the primary end point). A sample size of 6 per group (patients with hepatic impairment and HVs) is considered sufficient for hepatic impairment trials.

Safety, tolerability, PK, and exploratory end points were assessed using descriptive statistics. The effect of hepatic impairment on the PK of BI 685509 was assessed using an ANOVA model, and differences between BI 685509 treatment and placebo for systolic BP, diastolic BP, and MAP were assessed at day 28 using a linear regression model. PK parameters were calculated by noncompartmental analysis using Phoenix WinNonlin software (version 8.1; Pharsight Corporation, Mountain View, CA, USA).

### Trial registration

The trial was registered with ClinicalTrials.gov (NCT03842761).

## RESULTS

### Study participants and compliance

**FIGURE 1:**
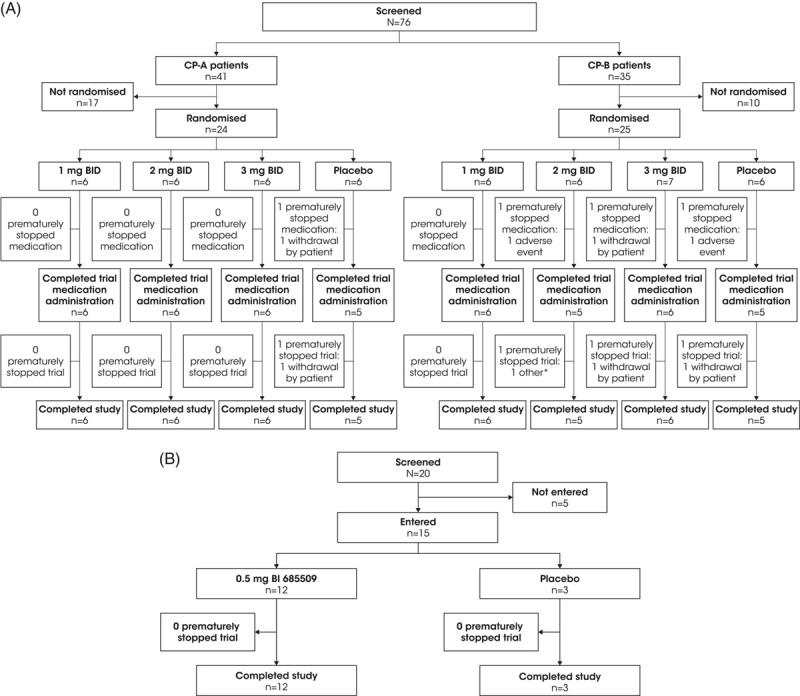
Participant disposition. (A) Child–Pugh class A and B patients; (B) healthy volunteers. *One CP-B patient in the 2 mg BID group was removed from the trial due to a serious AE of delirium. Abbreviations: AE, adverse event; BID, twice daily; CP, Child–Pugh.

In total, 96 participants were screened (CP-A, n = 41; CP-B, n = 35; HVs, n = 20; March 8, 2019, to February 3, 2021); of these, 24 patients with CP-A cirrhosis (n = 6 each per dose group and placebo), 25 patients with CP-B cirrhosis (n = 6 each per 1 mg BID and 2 mg BID; n = 7, 3 mg BID; n = 6, placebo), and 15 HVs (n = 12, BI 685509 0.5 mg once daily; n = 3, placebo) were treated (Figure 1). Participant characteristics and demographics were similar between dose groups and CP groups and between matched HVs and patients with CP-A and CP-B cirrhosis in the 1 mg BID dose group (Table 1). However, almost two-thirds of patients with CP-B cirrhosis were male, and the proportion of male patients varied between dose groups. Cirrhosis history (cause of cirrhosis, prior decompensation events) varied between patients with CP-A and CP-B cirrhosis: 7 patients with CP-A cirrhosis (29.2%) and 24 patients with CP-B cirrhosis (96.0%) had a prior decompensating event. The CP-B patient with no prior decompensation event was included in this arm because of high bilirubin levels at baseline (>3 mg/dL).

**TABLE 1:** Patient demographics, disease characteristics, and biomarkers

	CP-A cirrhosis					CP-B cirrhosis					Healthy volunteers		
Characteristic	1 mg BID (n=6)	2 mg BID (n=6)	3 mg BID (n=6)	Placebo (n=6)	Total (n=24)	1 mg BID (n=6)	2 mg BID (n=6)	3 mg BID (n=7)	Placebo (n=6)	Total (n=25)	0.5 mg QD (n=12)	Placebo (n=3)	Total (n=15)
Sex, male, n (%)	3 (50.0)	2 (33.3)	4 (66.7)	2 (33.3)	11 (45.8)	1 (16.7)	4 (66.7)	7 (100.0)	4 (66.7)	16 (64.0)	4 (33.3)	2 (66.7)	6 (40.0)
Race, n (%)													
White	6 (100.0)	6 (100.0)	6 (100.0)	6 (100.0)	24 (100.0)	6 (100.0)	6 (100.0)	7 (100.0)	6 (100.0)	25 (100.0)	10 (83.3)	3 (100.0)	13 (86.7)
Black or African-American	0	0	0	0	0	0	0	0	0	0	2 (16.7)	0	2 (13.3)
Age, y, mean (SD)	59.5 (6.0)	62.2 (5.2)	61.8 (7.3)	59.5 (6.0)	60.8 (6.2)	60.3 (4.3)	57.0 (5.0)	53.7 (8.5)	56.8 (8.1)	56.8 (6.8)	60.1 (6.7)	56.7 (8.3)	59.4 (6.9)
Bodyweight, kg, mean (SD)	82.0 (13.0)	85.1 (12.4)	93.5 (15.1)	81.8 (16.4)	85.6 (14.2)	90.9 (26.4)	88.9 (22.9)	112.4 (33.7)	97.2 (15.9)	98.0 (26.2)	82.9 (16.1)	103.9 (7.0)	87.1 (16.9)
BMI, kg/m^2^, mean (SD)	30.9 (5.1)	32.9 (5.7)	33.3 (5.7)	29.9 (5.3)	31.8 (5.3)	35.8 (10.2)	30.3 (5.9)	36.3 (9.6)	35.8 (5.0)	34.6 (8.0)	29.5 (6.01)	34.5 (2.9)	30.5 (5.8)
MELD, mean (SD)	8.2 (2.1)	7.3 (1.4)	7.7 (1.2)	8.0 (1.1)	7.8 (1.4)	10.5 (2.6)	9.0 (2.1)	8.7 (2.1)	10.8 (2.1)	9.7 (2.3)	–	–	–
Liver stiffness, kPa, mean (SD)	29.3 (10.7)	23.0 (10.9)	25.1 (1.9)	28.8 (8.3)	26.5 (8.6)	46.7 (24.1)	38.9 (16.4)	33.0 (22.1)	28.8 (15.2)	37.1 (19.8)	–	–	–
Patients with LSM > 20 kPa, n (%)	5 (83.3)	4 (66.7)	6 (100.0)	5 (83.3)	20 (83.3)	6 (100.0)	5 (83.3)	4 (57.1)	3 (50.0)	18 (72.0)	–	–	–
Patients with LSM > 25 kPa, n (%)	4 (66.7)	2 (33.3)	4 (66.7)	4 (66.7)	14 (58.3)	5 (83.3)	4 (66.7)	4 (57.1)	3 (50.0)	16 (64.0)	–	–	–
MAP, mm Hg, mean (SD)	95.5 (10.9)	100.8 (14.7)	99.2 (8.2)	93.1 (14.1)	97.2 (11.9)	90.9 (12.3)	89.7 (15.7)	95.2 (9.7)	79.6 (6.1)	89.1 (12.4)	–	–	–
Platelet count, 10^9^/L, mean (SD)	100.9 (19.3)	106.3 (32.3)	108.4 (30.4)	106.5 (31.9)	105.5 (27.2)	94.5 (18.4)	102.4 (31.3)	126.0 (42.5)	88.4 (39.3)	103.8 (35.7)	254.1 (41.4)	239.7 (118.5)	–
Patients with platelets <150 G/L, n (%)	6 (100.0)	5 (83.3)	6 (100.0)	6 (100.0)	23 (95.8)	6 (100.0)	6 (100.0)	5 (71.4)	5 (83.3)	22 (88.0)	0 (0.0)	1 (33.3)	1 (6.67)
Bilirubin, µmol/L, mean (SD)	12.9 (4.1)	13.9 (4.4)	12.5 (2.4)	15.2 (4.7)	13.6 (30.3)	22.1 (8.8)	17.7 (5.8)	17.6 (8.0)	23.8 (13.8)	20.2 (9.3)	8.8 (2.2)	9.4 (2.3)	–
Cirrhosis etiology, n (%)													
NAFLD	2 (33.3)	4 (66.7)	3 (50.0)	1 (16.7)	10 (41.7)	4 (66.7)	0	1 (14.3)	3 (50.0)	8 (32.0)	–	–	–
ALD	2 (33.3)	0	1 (16.7)	2 (33.3)	5 (20.8)	1 (16.7)	2 (33.3)	6 (85.7)	1 (16.7)	10 (40.0)	–	–	–
Chronic viral hepatitis	2 (33.3)	2 (33.3)	1 (16.7)	3 (50.0)	8 (33.3)	1 (16.7)	3 (50.0)	0	2 (33.3)	6 (24.0)	–	–	–
Other^a^	0	0	1 (16.7)	0	1 (4.2)	0	1 (16.7)	0	0	1 (4.0)	–	–	–

a Other etiologies: CP-A 3 mg BID, hemochromatosis; CP-B 2 mg BID, autoimmune hepatitis.

Abbreviations: ALD, alcohol-associated liver disease; BID, twice daily; CP, Child–Pugh; LSM, liver stiffness measurement; QD, once daily.

In patients with CP-A cirrhosis, prior decompensating events were ascites (n = 1, placebo), encephalopathy (n = 2, 1 mg BID; n = 1, placebo), variceal bleeding (n = 1 each per 3 mg BID and placebo), gastrointestinal bleeding (n=1, 3 mg BID), and elevated bilirubin (n = 1, placebo). In patients with CP-B cirrhosis, prior decompensating events were ascites (n = 1, 1 mg BID; n = 5 each per 2 mg BID and 3 mg BID; n = 4, placebo), encephalopathy (n = 1, 1 mg BID; n = 2, 2 mg BID; n = 3, placebo), and variceal bleeding (n = 4, 1 mg BID; n = 1 each per 2 mg BID, 3 mg BID, and placebo). Baseline liver stiffness values were >20 kPa for 83.3% and 72.0% of patients with CP-A and CP-B cirrhosis, respectively, and were >25 kPa for 58.3% and 64.0% of patients with CP-A and CP-B cirrhosis, respectively.

During the trial, 1 patient with CP-A cirrhosis (4.2%, placebo) and 3 patients with CP-B cirrhosis (12.0%; n = 1 each per 2 mg BID, 3 mg BID, and placebo) discontinued trial medication: 2 due to an AE (CP-B, n = 1 each per 3 mg BID [delirium] and placebo [hematemesis, non-variceal]) and 2 due to withdrawal by participant (CP-A: n = 1, placebo; CP-B: n = 1, 3 mg BID). All treated patients with CP-A and CP-B cirrhosis and matched HVs were included in the biomarker set and the ECG plasma concentration set, and all patients with CP-A and CP-B cirrhosis and HVs on active treatment were included in the PK parameter analysis.

Important protocol deviations were reported for 4 patients with CP-A cirrhosis, 8 patients with CP-B cirrhosis, and 1 HV but did not lead to patient exclusion from the analysis sets. Good compliance with trial medication (80–120%) was recorded for most patients, with exceptions for 2 patients with CP-A cirrhosis (n = 1, 1 mg BID [67.0% on days 1 and 4–9]; n = 1, placebo [50.0% on days 1 and 4–9]) and 2 patients with CP-B cirrhosis (n = 1, 2 mg BID [50% on days 10–15]; n = 1, placebo [75.0% on days 10–15]).

### Safety

Investigator-defined, drug-related AEs (primary end point) were reported for 2 patients with CP-A cirrhosis (8.3%: n = 1, 3 mg BID; n = 1, placebo) and 5 patients with CP-B cirrhosis (20.0%: n = 3, 2 mg BID; n = 2, 3 mg BID) (Table 2). Five patients were reported with orthostatic intolerance (n = 1, CP-A 3 mg BID; n = 2 each per CP-B 2 mg BID and CP-B 3 mg BID), 1 patients with CP-A cirrhosis receiving placebo was reported with dizziness, and 1 patients with CP-B cirrhosis in the 2 mg BID dose group was reported with diarrhea.

**TABLE 2:** Summary of treatment-emergent adverse events according to cirrhosis Child–Pugh class and BI 685509 dose

	CP-A cirrhosis					CP-B cirrhosis				
TEAE, n (%)	1 mg BID (n=6)	2 mg BID (n=6)	3 mg BID (n=6)	Placebo (n=6)	Total (n=24)	1 mg BID (n=6)	2 mg BID (n=6)	3 mg BID (n=7)	Placebo (n=6)	Total (n=25)
Any TEAE	1 (16.7)	0	1 (16.7)	1 (16.7)	3 (12.5)	1 (16.7)	4 (66.7)	2 (28.6)	1 (16.7)	8 (32.0)
Investigator-defined drug-related AEs	0	0	1 (16.7)	1 (16.7)	2 (8.3)	0	3 (50.0)	2 (28.6)	0	5 (20.0)
Orthostatic intolerance	0	0	1 (16.7)	0	1 (4.2)	0	2 (33.3)	2 (28.6)	0	4 (16.0)
Dizziness	0	0	0	1 (16.7)	1 (4.2)	0	0	0	0	0
Diarrhea	0	0	0	0	0	0	1 (16.7)	0	0	1 (4.0)
Severe AEs										
Serious AEs	0	0	0	0	0	1 (16.7)	1 (16.7)	0	1 (16.7)	3 (12.0)
AEs leading to discontinuation of study drug	0	0	0	0	0	0	1 (16.7)	0	1 (16.7)	2 (8.0)

Abbreviations: AE, adverse event; BID, twice daily; CP, Child–Pugh; TEAE, treatment-emergent adverse event.

TEAEs were reported for 3 patients with CP-A cirrhosis (12.5%) and 8 patients with CP-B cirrhosis (32.0%); no HVs reported TEAEs (Table 2). The most frequent TEAEs in patients with CP-A cirrhosis were orthostatic intolerance (n = 1, 3 mg BID), dizziness (n = 1, placebo), and influenza-like illness (n = 1, 1 mg BID). The most frequent TEAE in patients with CP-B cirrhosis was orthostatic intolerance (n = 4: n = 2 each per 2 mg BID and 3 mg BID). No serious AEs or AEs leading to discontinuation of the trial drug were reported in patients with CP-A cirrhosis. Three serious AEs were reported in patients with CP-B cirrhosis (n = 1 each per ascites, 1 mg BID; delirium, 2 mg BID; hematemesis [non-variceal], placebo), 2 of which led to discontinuation of the trial drug, but none of these AEs were considered drug-related.

No relevant changes from baseline in systolic BP, diastolic BP, HR, bodyweight (secondary end points), MAP, hip circumference, or waist circumference were seen in patients with CP-A or CP-B cirrhosis at day 28 or in HVs (Table 3). At day 28, no significant difference was seen between patients treated with BI 685509 and patients who received a placebo for systolic BP, diastolic BP, or MAP (*p* > 0.05). However, over the course of the study, decreases from baseline in 1 hour postdose systolic BP and 1 hour postdose diastolic BP were seen in patients treated with BI 685509 compared with placebo, although no clear dose relationship was seen in either patients with CP-A or CP-B cirrhosis (Supplemental Figures S1A–D, http://links.lww.com/HC9/A535). Overall, HR increased from baseline at 1 hour postdose in patients with CP-A cirrhosis receiving 3 mg BID BI 685509 and patients with CP-B cirrhosis receiving 2 mg and 3 mg BID BI 685509 (Supplemental Figures S1E and F, http://links.lww.com/HC9/A535).

**TABLE 3:** Change from baseline in secondary safety end points at day 28

	CP-A cirrhosis				CP-B cirrhosis			
	1 mg BID (n=6)	2 mg BID (n=6)	3 mg BID (n=6)	Placebo (n=6)	1 mg BID (n=6)	2 mg BID (n=6)	3 mg BID (n=7)	Placebo (n=6)
Change from baseline at day 28, mean (SD)								
Systolic BP, mm Hg	−7.7 (13.2)	−24.3 (23.8)	−4.7 (24.8)	−7.8 (13.4)^a^	−14.2 (13.8)^a^	3.4 (4.2)^a^	−12.0 (22.6)^b^	4.0 (11.5)^a^
Diastolic BP, mm Hg	0.5 (15.2)	−7.7 (16.5)	−0.2 (10.5)	−0.8 (4.9)^a^	−8.6 (13.2)^a^	−1.6 (6.6)^a^	−6.8 (12.7)^b^	2.8 (10.4)^a^
Heart rate, bpm	−6.5 (12.5)	−9.7 (14.2)	3.5 (5.9)	0.2 (4.1)^a^	−4.8 (17.7)^a^	0.0 (5.9)^a^	−9.3 (9.9)^b^	−5.2 (3.4)^a^
Bodyweight, kg	−0.2 (1.8)	−0.3 (2.7)	−0.2 (2.0)	0.5 (1.5)^a^	1.4 (2.5)^a^	−0.1 (1.7)^a^	−1.3 (2.5)^b^	−2.3 (4.0)^a^
Hip circumference, cm	0.8 (2.2)	0.5 (6.1)	2.7 (6.1)	1.2 (3.4)^a^	1.8 (4.6)	2.0 (9.2)^a^	−0.1 (4.3)^b^	−3.3 (3.4)^a^
Waist circumference, cm	0.1 (2.3)	−0.2 (4.5)	−0.7 (5.0)	−0.2 (2.9)^a^	−0.2 (4.7)	0.4 (5.1)^a^	5.0 (8.7)^b^	−4.2 (3.5)^a^
Absolute values at day 28, mean (SD)								
Systolic BP, mm Hg	132.2 (13.6)	120.2 (16.8)	135.7 (13.5)	125.0 (28.7)^a^	115.2 (14.7)^a^	128.8 (13.7)^a^	120.3 (14.4)^b^	112.0 (6.9)^a^
Diastolic BP, mm Hg	73.8 (8.5)	71.3 (13.9)	78.5 (15.7)	75.2 (12.8)^a^	61.6 (3.8)^a^	76.4 (10.4)^a^	69.7 (9.4)^b^	66.6 (7.2)^a^
Heart rate, bpm	64.2 (5.9)	63.0 (9.2)	73.7 (6.4)	60.0 (5.8)^a^	65.8 (9.8)^a^	68.8 (8.8)^a^	64.5 (6.8)^b^	69.8 (7.6)^a^
Bodyweight, kg	81.9 (11.7)	84.6 (13.3)	93.1 (16.4)	77.3 (11.6)^a^	91.2 (28.3)^a^	88.1 (24.7)^a^	113.4 (34.9)^b^	96.9 (21.6)^a^
Hip circumference, cm	105.2 (8.5)	113.4 (10.2)	116.3 (15.0)	104.8 (10.4)^a^	115.7 (18.0)	110.0 (13.1)^a^	122.5 (23.6)^b^	114.6 (18.4)^a^
Waist circumference, cm	101.7 (11.7)	104.5 (15.2)	115.3 (14.0)	94.4 (10.2)^a^	109.7 (20.0)	105.4 (15.0)^a^	119.2 (20.6)^b^	107.6 (16.4)^a^

a n=5.

b n=6.

Abbreviations: BID, twice daily; CP, Child–Pugh.

Overall, 5 patients were reported with orthostatic dysregulation (n = 1, CP-A 3 mg BID; n = 2 each per CP-B 2 mg BID and 3 mg BID); no matched HVs were reported with orthostatic dysregulation. For CP-A 1 mg BID and CP-B 2 mg BID patients, systolic BP after standing up tended to decrease for all 1 hour postdose measurements compared with predose measurements, and pulse rate tended to increase 1 hour postdose compared with predose measurements (Supplemental Figure S2, http://links.lww.com/HC9/A535). Notable ECG findings were reported for HR in 2 patients with CP-A cirrhosis (2 mg BID, 45.2–51.1% decrease; 3 mg BID, 46.1% increase), and an increased QT interval (>500 msec) was reported for 1 CP-B patient on day 1 of treatment (1 mg BID; QT interval 502 msec). No clinically relevant findings were reported for HVs. A new onset of maximum QT interval corrected by Frederica's formula (QTcF) interval (QTcF interval >500 msec or change from baseline of >60 msec at any time of treatment) was observed in 7 patients with CP-A cirrhosis (n = 1, 1 mg BID; n = 3, 2 mg BID; n = 2, 3 mg BID; and n = 1, placebo) and 9 patients with CP-B cirrhosis (n = 3, 1 mg BID; n = 5, 2 mg BID; and n = 1, 3 mg BID). In addition, only BI 685509-treated patients had an increase from baseline in QTcF of ≥30 msec or QT of ≥60 msec, although no dose relationship was observed. The time profiles of QTcF and HR changes from baseline were similar between patients with CP-A and CP-B cirrhosis; no notable findings were observed in patients receiving placebo.

Two patients with CP-A cirrhosis progressed to CP-B during treatment (n = 1 each per 1 mg BID and 3 mg BID), with the patient in the 3 mg BID dose group returning to CP-A at follow-up. Two patients with CP-B cirrhosis were reported with a temporary shift to CP-A (n = 1 each per 1 mg BID and placebo), and 1 patient reported a temporary shift to CP-C (1 mg BID) due to decreased serum albumin. All patients reporting a shift in CP classification continued treatment.

During treatment, 7 patients with CP-A cirrhosis and 12 patients with CP-B cirrhosis (no HVs) had slightly raised direct bilirubin levels (maximum values: 0.43–0.76 mg/dL and 0.44–1.24 mg/dL, respectively). Of these, only 2 episodes were considered possibly clinically significant (both in patients with CP-B cirrhosis; maximum values: 1.14 mg/dL and 1.24 mg/dL). Furthermore, during treatment, 12 patients with CP-A cirrhosis, 11 patients with CP-B cirrhosis, and 1 HV had slightly raised alkaline phosphatase levels (maximum values: 119–214 U/L, 118–203 U/L, and 151 U/L, respectively); none of these episodes were considered clinically significant.

### PK outcomes

**FIGURE 2:**
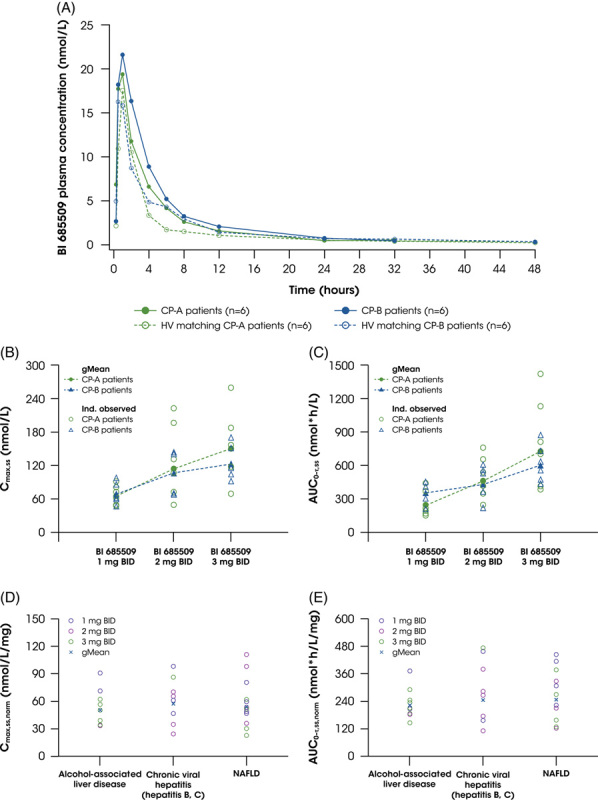
Pharmacokinetic outcomes of BI 685509 in plasma. (A) Plasma concentration–time profiles of BI 685509 in Child–Pugh class A and B patients and matched healthy volunteers after single-dose administration. Plasma C_max,ss_ (B) and AUC_0–τ,ss_ (C) of BI 685509 across dose groups and Child–Pugh classifications. Dose-normalized C_max,ss_ (D) and AUC_0–τ,ss_ (E) across cirrhosis etiologies. Abbreviations: BID, twice daily; CP, Child -Pugh; gMean, geometric mean; HV, healthy volunteer.

Following single and multiple oral administrations of BI 685509 in patients with mild or moderate hepatic impairment, the drug was rapidly absorbed (median t_max(ss)_, 0.5–2.0 hours) and exposure (C_max(ss)_ and AUC_(ss)_) of BI 685509 increased with dose (Table 4). Plasma concentration–time profiles were similar after single-dose administration on day 1 between patients with CP-A cirrhosis, patients with CP-B cirrhosis, and matched HVs (Figure 2A) and after multiple doses on days 28–31 between patients with CP-A and CP-B cirrhosis; however, concentrations of BI 685509 were higher in patients with CP-A and CP-B cirrhosis compared with matched HVs.

**TABLE 4:** Pharmacokinetic parameters of BI 685509 treatment

	CP-A cirrhosis			CP-B cirrhosis			Healthy volunteers	
PK parameter, gMean (gCV, %)	1 mg BID (n=6)	2 mg BID (n=6)	3 mg BID (n=6)	1 mg BID (n=6)	2 mg BID (n=6)	3 mg BID (n=7)	CP-A matched (n=6)	CP-B matched (n=6)
AUC_0–24_, nmol·h/L	87.6 (45.8)	172 (51.8)	328 (35.5)	110 (49.0)	176 (49.0)	319 (10.4)	59.5 (17.9)	84.4 (47.8)
AUC_0–tz_, nmol·h/L	95.2 (49.7)	197 (61.2)	367 (37.8)	120 (48.3)	193 (45.5)	363 (11.1)	67.8 (20.5)	94.6 (49.7)
AUC_0–∞_, nmol·h/L	101 (50.4)	203 (61.2)	373 (37.5)	126 (52.1)^a^	198 (43.1)	378 (13.0)	73.7 (19.4)	102 (47.4)
AUC_τ,ss_, nmol·h/L	245 (43.6)	464 (43.6)	729 (55.9)	357 (33.5)^a^	434 (43.7)^a^	604 (26.6)^a^	–	–
C_max_, nmol/L	25.5 (29.2)	53.6 (20.9)	90.5 (20.4)	22.7 (49.9)	50.6 (26.3)	63.7 (25.3)	18.5 (24.0)	23.4 (66.2)
C_max,ss_, nmol/L	64.7 (27.3)	114 (63.3)	150 (48.6)	68.0 (30.4)^a^	106 (41.0)^a^	122 (23.4)^a^	–	–
t_max_, h	0.750 (0.500–2.00)	0.500 (0.500–1.00)	0.750 (0.500–1.00)	0.750 (0.500–1.00)	0.500 (0.500–0.500)	2.00 (0.500–2.00)	1.00 (0.500–1.12)	0.750 (0.500–6.00)
t_max,ss_, h	0.500 (0.250–2.00)	0.767 (0.250–2.000)	0.750 (0.500–1.08)	1.00 (0.500–1.00)^a^	0.500 (0.500–1.00)^a^	1.50 (0.500–2.00)^a^	–	–
t_½_, h	10.2 (62.0)	10.9 (61.2)	12.0 (27.9)	10.4 (39.4)^a^	9.27 (56.2)	12.7 (65.3)	15.7 (25.6)	13.2 (66.2)
t_½,ss_, h	12.2 (54.8)	15.4 (35.3)	12.2 (22.6)	13.8 (93.6)^b^	10.8 (75.7)^a^	14.4 (116)^a^	–	–
PTF, %	279 (35.3)	257 (43.4)	206 (32.0)	183 (26.2)^a^	262 (24.5)^a^	197 (47.4)^a^	–	–
MRT_ex_, h	9.45 (60.2)	10.3 (61.8)	9.69 (25.8)	10.7 (22.5)^a^	8.78 (42.7)	12.4 (40.6)	13.4 (29.8)	11.8 (59.9)
MRT_ex,ss_, h	11.1 (52.0)	11.0 (27.2)	12.1 (45.5)	13.7 (38.6)^b^	8.59 (32.1)^a^	13.9 (79.2)^a^	–	–
CL/F, mL/min	141 (50.4)	141 (61.2)	153 (37.5)	114 (52.1)^a^	144 (43.1)	151 (13.0)	194 (19.4)	140 (47.4)
CL/F_ss_, mL/min	117 (43.6)	123 (43.6)	118 (55.9)	80.2 (33.5)^a^	132 (43.7)^a^	142 (26.6)^a^	–	–
V_z_/F, L	125 (68.7)	133 (69.8)	159 (41.1)	103 (92.1)^a^	116 (96.0)	167 (54.8)	264 (22.2)	160 (78.9)
V_z_/F_ss_, L	123 (61.3)	164 (61.5)	124 (77.7)	84.4 (73.3)^b^	124 (119)^a^	177 (89.0)^a^	–	–
C_min,ss_, nmol/L	6.65 (71.1)	13.7 (61.2)	24.0 (36.3)	12.1 (89.9)^a^	10.0 (74.8)^a^	18.6 (70.6)^a^	–	–
C_avg_, nmol/L	20.4 (43.6)	38.6 (43.6)	60.8 (55.9)	29.7 (33.5)^a^	36.1 (43.7)^a^	50.4 (26.6)^a^	–	–
fe_0–24_, %	1.21 (30.8)	0.916 (78.3)	0.727 (81.9)	1.33 (60.4)	1.45 (55.4)^a^	1.33 (63.4)	0.601 (15.0)	0.462 (56.6)
fe_0–12,ss_, %	1.07 (52.0)	1.34 (37.7)^a^	1.31 (51.4)	2.52 (35.9)^b^	1.69 (55.1)^a^	0.958 (52.3)^a^	–	–
CL_R, 0–24_, mL/min	1.97 (66.1)	1.52 (38.8)	1.27 (97.4)	1.73 (38.0)	2.46 (27.4)^a^	2.38 (53.5)	1.45 (18.4)	0.731 (82.2)^a^
CL_R, 0–12, ss_, mL/min	1.25 (25.6)	1.56 (52.6)^a^	1.54 (62.1)	–	2.24 (21.1)^a^	1.31 (71.9)^b^	–	–

a n-1.

b n-2.

Abbreviations: BID, twice daily; CL/F, apparent clearance; CL_R_, renal clearance; CP, Child–Pugh; fe, fraction of dose excreted; gCV, geometric coefficient of variation; gMean, geometric mean; MRT, mean residence time; PK, pharmacokinetic; PTF, peak-trough fluctuation; t_max_, time to peak plasma concentration; V_Z_/F, apparent volume of distribution.

The PK of multiple doses of BI 685509 were consistent with the single-dose results and between patient groups (Table 4). The apparent clearance (CL/F and CL/F_ss_), volume of distribution (V_z_ and V_z_/F_ss_), terminal elimination half-life (t_1/2_ and t_1/2,ss_), and renal clearance were comparable between patients with CP-A and CP-B cirrhosis (Table 4). Overall, estimates of AUC_0–tz_ and C_max_ and the geometric means of AUC_τ,ss_ and C_max,ss_ were similar between patients with CP-A and CP-B cirrhosis (Figure 2B and C; Table 4); both parameters at steady state were slightly lower for CP-B 3 mg BID, and AUC_τ,ss_ was slightly higher for CP-B 1 mg BID, compared with patients with CP-A cirrhosis. Dose-normalized C_max,ss_ and AUC_τ,ss_ values were similar between the different cirrhosis etiologies on day 28 (Figure 2D and E).

A comparison of patients with hepatic impairment with matched HVs showed that exposure was ~40% and ~27% higher in patients with CP-A and CP-B cirrhosis, respectively. The CL/F and V_Z_/F were lower in patients compared with matched HVs, whereas the fraction of dose excreted (fe_0–24_) and renal clearance from 0 to 24 hours (CL_R,0–24_) were higher in patients. The t_1/2_ was comparable between patients and their matched HVs.

### Efficacy outcomes

**FIGURE 3:**
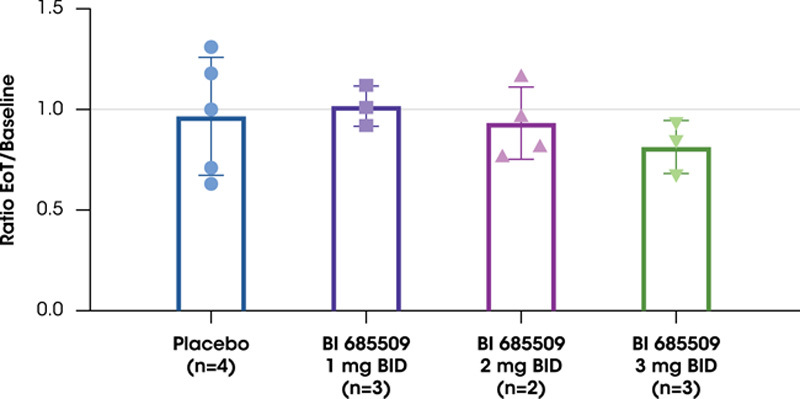
Spleen stiffness in patients with Child -Pugh class A cirrhosis. Data are presented as mean±SD, with individual values represented. Abbreviations: BID, twice daily; EoT, end of treatment.

The change from baseline in spleen stiffness was inconclusive due to a high proportion of invalid measurements (31%) and technical and biological variability (Supplemental Table S2, http://links.lww.com/HC9/A535). Although no significant changes in median spleen stiffness occurred over time, the available paired end-of-treatment/baseline spleen stiffness ratios were all ≤1 for all patients with CP-A cirrhosis (Figure 3).

**FIGURE 4:**
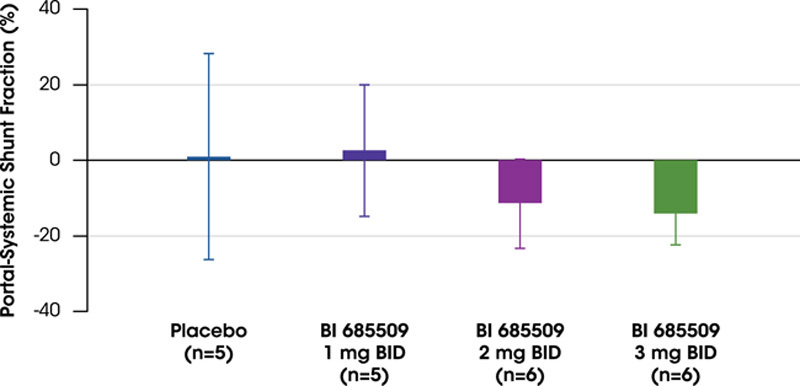
Change in portal–systemic shunt fraction in patients with Child–Pugh class A cirrhosis as measured by the HepQuant SHUNT test. Data are presented as mean±SD. Abbreviations: BID, twice daily.

The 2 highest doses of BI 685509 treatment decreased the portal–systemic shunt fraction of the HepQuant SHUNT test from baseline to day 27 (Figure 4; Supplemental Table S2, http://links.lww.com/HC9/A535). The mean (SD) percentage changes from baseline were 2.36% (17.43) for 1 mg BID, −11.15% (11.89) for 2 mg BID, −14.03% (8.35) for 3 mg BID, and 0.99% (27.31) for placebo. No clinically relevant change from baseline was seen for disease severity index and STAT in any dose group or with placebo (Supplemental Table S2, http://links.lww.com/HC9/A535); however, disease severity index was decreased by 3 mg BID BI 685509 (−6.96% [7.19]; p = 0.06 vs. baseline), and STAT was decreased by both 2 mg BID and 3 mg BID ( −10.62% [25.03] and −10.16% [41.25]), respectively.

At baseline, liver stiffness was higher in patients with CP-B cirrhosis (median 28.8–46.7 kPa) compared with patients with CP-A cirrhosis (median 23.0–29.3 kPa; Table 1), and no treatment effect was seen for liver stiffness, fat content, or Pro-C3 levels (Supplemental Table S2, http://links.lww.com/HC9/A535); large interindividual variability was seen for liver stiffness values.

Overall, no relevant differences between patient groups or treatment groups or relevant changes from baseline were observed for any assessed biomarkers, composite scores, or patient-reported outcomes (Supplemental Table S2, http://links.lww.com/HC9/A535).

## DISCUSSION

### Safety

In this Phase Ib trial, BI 685509 was generally well tolerated, with 2 AEs leading to treatment discontinuation and 3 serious AEs, restricted to patients with CP-B cirrhosis and assessed as not drug-related. The frequency of TEAEs and drug-related AEs was higher in patients with CP-B cirrhosis (32.0% and 20.0%, respectively) compared with patients with CP-A cirrhosis (12.5% and 8.3%, respectively), with orthostatic intolerance being the most frequently reported drug-related AE (n = 1 each per CP-A 3 mg BID, CP-B 2 mg BID, and CP-B 3 mg BID). Decreases in systolic and diastolic BP and an increase in HR were seen in patients receiving BI 685509 compared with placebo; however, no dose relationship was observed in either patient group. This could be a benefit of the mechanism of action of this therapy compared with NSBBs, with which hypotension often occurs in patients with CP-B cirrhosis.^25^ However, drug-related orthostatic dysregulation occurred in 5 patients receiving BI 685509 (n = 1, CP-A 3 mg BID; n = 2 each per CP-B 2 mg BID and 3 mg BID). Although no relevant effects of BI 685509 on vital signs were identified during orthostatic testing, trends of decreased standing systolic BP and increased pulse rate were observed in CP-A 1 mg BID and CP-B 2 mg BID patients. Additionally, increases in HR and in QTcF and QT intervals were observed. However, only a limited number of data points were available in the concentration–QTcF analysis, such that the results may be interpreted with caution. Overall, no relevant cardiac arrhythmias occurred, and the clinical relevance of QT interval changes is currently unknown. Nonetheless, these changes led to the identification of a potential risk of QT prolongation following BI 685509 treatment, the implementation of risk minimization strategies, and the inclusion of regular QT assessments in subsequent clinical studies.

### PK outcomes

As BI 685509 is expected to be primarily eliminated by the liver (unpublished data), it is anticipated that hepatic impairment may lower the overall clearance of BI 685509, leading to higher systemic exposure. Indeed, when compared with matched HVs, apparent clearance was ~27% and ~19% lower in CP-A and CP-B patients, respectively. In addition, BI 685509 total exposure in patients with hepatic impairment (AUC_0–tz_) was ~30–40% higher compared with matched HVs. Although exposure was higher in patients with hepatic impairment relative to matched HVs, it is anticipated that the severity of hepatic impairment will affect the clearance of the compound by the liver. However, clearance and the overall exposure of BI 685509 in patients with mild (CP-A) and moderate (CP-B) hepatic impairment were similar. Currently, the effect of the severity of hepatic impairment on the PK of BI 685509 remains unclear and will be explored further in the future.

The PK and safety data obtained from this Phase Ib study informed the study design and BI 685509 dose selection for 2 ongoing Phase II studies in patients with cirrhosis and clinically significant PH (NCT05161481 and NCT05282121).

### Efficacy outcomes

In this study, treatment with the 2 highest doses of BI 685509 dose-dependently decreased mean portal–systemic shunt fraction. The HepQuant SHUNT test uses the hepatic uptake of cholate from the systemic and portal circulations to assess liver function and portal blood flow.^26^ Progressive liver disease is associated with a decrease in cholate clearance and an increase in portal–systemic shunting, thereby allowing the test to identify the risk of complications in patients with compensated cirrhosis.^26^ This test is also capable of the early, noninvasive detection of PH, with a reliability similar to that of HVPG.^27^ However, while HVPG remains the gold standard method for assessing PH, and its use is encouraged in clinical trials, it is invasive and requires considerable resources and expertise.^28^ Therefore, HepQuant SHUNT and other noninvasive biomarkers (eg, spleen stiffness measurements^28^) may complement HVPG in the short term and perhaps replace it in the future.

Although spleen stiffness may identify a portal pressure decrease in response to NSBBs,^29^ the current study was the first clinical trial to use spleen stiffness as assessed by a dedicated vibration-controlled transient elastography device (ie, FibroScan Expert 630 Professional) as a surrogate end point for PH. However, spleen stiffness measurements in the present study showed high intraindividual variability and were invalid and inconclusive in many patients. There are several reasons why the results had a high degree of variability. First, spleen stiffness measurements can have lower repeatability than liver stiffness measurements.^30^ While the device used for this study (FibroScan 630 Expert) uses a novel 100 Hz probe specifically designed for spleen stiffness measurements,^31^ the measurement depth of the probe (M-Probe) is limited to between 25 and 65 mm. Notably, since the mean body mass index was >30 kg/m^2^ (the definition of obesity) for each patient group and for HVs in this study, there tended to be a long skin-to-spleen distance. This increased distance may make the probe nonoptimal for use in people with obesity.^32^ Finally, spleen stiffness measurements can depend on operator experience. The disruption caused by the COVID-19 pandemic made training for this novel device difficult, possibly resulting in less-experienced investigators and, therefore, more variable measurements. Further exploration of spleen stiffness as a noninvasive biomarker for PH in future clinical trials is warranted, including the optimization of probe use for patients with a high body mass index.

### Limitations

A limitation of this study was the short treatment duration, with only 12 days spent on the dose-escalated maintenance dose. This may explain why biomarkers of liver function and liver stiffness measurements remained unchanged; however, the primary end point of this study was to assess the safety and tolerability of BI 685509 and not to explore efficacy end points. In addition, the low number of included patients could have influenced the power of the exploratory outcomes. Furthermore, although most patients with CP-A cirrhosis did not show any clinical evidence of decompensation, some patients may have experienced self-limiting and resolved decompensation events ≥6 weeks before screening. During the study, some patients with CP-A cirrhosis may have had disease characteristics similar to patients with CP-B cirrhosis; however, only 2 patients with CP-A cirrhosis progressed to clinically evident CP-B during treatment. In addition, phosphatidylethanol blood alcohol testing was not conducted to confirm alcohol abstinence throughout the trial. However, participants were excluded if they had ongoing chronic alcohol or drug use.

Although this trial did not show reductions in markers of fibrosis, preclinical studies of sGC modulation in various models have shown that promoting sGC activity and, thus increasing cyclic guanosine monophosphate production, can both decrease portal pressure and exert antifibrotic effects.^19–21,33,34^ Phase II studies of BI 685509 in patients with cirrhosis have been designed and are ongoing to investigate the efficacy of BI 685509 to ameliorate PH (ie, to decrease HVPG).

## CONCLUSIONS

This Phase Ib clinical trial examined the safety, tolerability, and PK of BI 685509, an sGC activator, in patients with CP-A or CP-B cirrhosis and HVs. This study demonstrated that multiple rising doses of BI 685509 were generally well tolerated in patients with mild or moderate hepatic impairment, with 3 serious AEs reported (all in patients with CP-B cirrhosis) and none considered to be drug-related. The PK and exposure of BI 685509 were similar between patient groups with mild (ie, CP-A) versus moderate (ie, CP-B) hepatic impairment and across different cirrhosis etiologies. Exposure of BI 685509 in matched HVs was lower compared with patients with hepatic impairment. While paired spleen stiffness measurements all decreased with BI 685509 treatment, the high failure rate of the technique, likely due to the prevalence of obesity in this population, limits this conclusion. The HepQuant SHUNT test results suggest that high doses of BI 685509 reduce portal–systemic shunting, indicating improved hepatic function. Thus, this trial supports further evaluation of BI 685509 for the treatment of patients with cirrhosis and PH.

## Supplementary Material

SUPPLEMENTARY MATERIAL

## References

[1] GBD 2017 Cirrhosis Collaborators. The global, regional, and national burden of cirrhosis by cause in 195 countries and territories, 1990-2017: A systematic analysis for the Global Burden of Disease Study 2017. Lancet Gastroenterol Hepatol. 2020;5:245–266. 31981519 10.1016/S2468-1253(19)30349-8 PMC7026710

[2] Garcia-Tsao G Abraldes JG Berzigotti A Bosch J. Portal hypertensive bleeding in cirrhosis: Risk stratification, diagnosis, and management: 2016 practice guidance by the American Association for the Study of Liver Diseases. Hepatology. 2017;65:310–335. 27786365 10.1002/hep.28906

[3] Groszmann RJ Garcia-Tsao G Bosch J Grace ND Burroughs AK Planas R. Beta-blockers to prevent gastroesophageal varices in patients with cirrhosis. N Engl J Med. 2005;353:2254–2261. 16306522 10.1056/NEJMoa044456

[4] Ripoll C Groszmann R Garcia-Tsao G Grace N Burroughs A Planas R. Hepatic venous pressure gradient predicts clinical decompensation in patients with compensated cirrhosis. Gastroenterology. 2007;133:481–488. 17681169 10.1053/j.gastro.2007.05.024

[5] Zipprich A Garcia-Tsao G Rogowski S Fleig WE Seufferlein T Dollinger MM. Prognostic indicators of survival in patients with compensated and decompensated cirrhosis. Liver Int. 2012;32:1407–1414. 22679906 10.1111/j.1478-3231.2012.02830.x PMC3713489

[6] de Franchis R Bosch J Garcia-Tsao G Reiberger T Ripoll C. Baveno VII - Renewing consensus in portal hypertension. J Hepatol. 2022;76:959–974. 35120736 10.1016/j.jhep.2021.12.022 PMC11090185

[7] Abraldes JG Trebicka J Chalasani N D’Amico G Rockey DC Shah VH. Prioritization of therapeutic targets and trial design in cirrhotic portal hypertension. Hepatology. 2019;69:1287–1299. 30318607 10.1002/hep.30314 PMC11090176

[8] Ge PS Runyon BA. Treatment of patients with cirrhosis. N Engl J Med. 2016;375:767–777. 27557303 10.1056/NEJMra1504367

[9] de Brito Nunes M Knecht M Wiest R Bosch J Berzigotti A. Predictors and management of post-banding ulcer bleeding in cirrhosis: A systematic review and meta-analysis. Liver Int. 2023;43:1644–1653. 37222256 10.1111/liv.15621

[10] Wiest R Groszmann RJ. The paradox of nitric oxide in cirrhosis and portal hypertension: Too much, not enough. Hepatology. 2002;35:478–491. 11826425 10.1053/jhep.2002.31432

[11] Derbyshire ER Marletta MA. Structure and regulation of soluble guanylate cyclase. Annu Rev Biochem. 2012;81:533–559. 22404633 10.1146/annurev-biochem-050410-100030

[12] Budworth J Meillerais S Charles I Powell K. Tissue distribution of the human soluble guanylate cyclases. Biochem Biophys Res Commun. 1999;263:696–701. 10512742 10.1006/bbrc.1999.1444

[13] Sandner P Zimmer DP Milne GT Follmann M Hobbs A Stasch JP. Soluble guanylate cyclase stimulators and activators. Handb Exp Pharmacol. 2021;264:355–394. 30689085 10.1007/164_2018_197

[14] Schaffner D Lazaro A Deibert P Hasselblatt P Stoll P Fauth L. Analysis of the nitric oxide-cyclic guanosine monophosphate pathway in experimental liver cirrhosis suggests phosphodiesterase-5 as potential target to treat portal hypertension. World J Gastroenterol. 2018;24:4356–4368. 30344420 10.3748/wjg.v24.i38.4356 PMC6189851

[15] Sarela AI Mihaimeed FM Batten JJ Davidson BR Mathie RT. Hepatic and splanchnic nitric oxide activity in patients with cirrhosis. Gut. 1999;44:749–753. 10205218 10.1136/gut.44.5.749 PMC1727519

[16] Thimgan MS Yee HF Jr. Quantitation of rat hepatic stellate cell contraction: Stellate cells’ contribution to sinusoidal resistance. Am J Physiol. 1999;277:G137–G143. 10409160 10.1152/ajpgi.1999.277.1.G137

[17] Elpek G. Cellular and molecular mechanisms in the pathogenesis of liver fibrosis: An update. World J Gastroenterol. 2014;20:7260–7276. 24966597 10.3748/wjg.v20.i23.7260 PMC4064072

[18] Hall KC Bernier SG Jacobson S Liu G Zhang PY Sarno R. sGC stimulator praliciguat suppresses stellate cell fibrotic transformation and inhibits fibrosis and inflammation in models of NASH. Proc Natl Acad Sci USA. 2019;116:11057–11062. doi:10.1073/pnas.1821045116 31085647 PMC6561202

[19] Sandner P Stasch JP. Anti-fibrotic effects of soluble guanylate cyclase stimulators and activators: A review of the preclinical evidence. Respir Med. 2017;122(suppl 1):S1–9. 28341058 10.1016/j.rmed.2016.08.022

[20] Schwabl P Brusilovskaya K Supper P Bauer D Königshofer P Riedl F. The soluble guanylate cyclase stimulator riociguat reduces fibrogenesis and portal pressure in cirrhotic rats. Sci Rep. 2018;8:9372. 29921982 10.1038/s41598-018-27656-y PMC6008436

[21] Xiao J Jin C Liu Z Guo S Zhang X Zhou X. The design, synthesis, and biological evaluation of novel YC-1 derivatives as potent anti-hepatic fibrosis agents. Org Biomol Chem. 2015;13:7257–7264. 26055070 10.1039/c5ob00710k

[22] Stasch JP Schlossmann J Hocher B. Renal effects of soluble guanylate cyclase stimulators and activators: A review of the preclinical evidence. Curr Opin Pharmacol. 2015;21:95–104. 25645316 10.1016/j.coph.2014.12.014

[23] Thoonen R Cauwels A Decaluwe K Geschka S Tainsh RE Delanghe J. Cardiovascular and pharmacological implications of haem-deficient NO-unresponsive soluble guanylate cyclase knock-in mice. Nat Commun. 2015;6:8482. 26442659 10.1038/ncomms9482 PMC4699393

[24] Reiberger T Schwabl P Trauner M Peck-Radosavljevic M Mandorfer M. Measurement of the hepatic venous pressure gradient and transjugular liver biopsy. J Vis Exp. 2020;e58819. doi:10.3791/58819 32628153

[25] Reiberger T Mandorfer M. Beta adrenergic blockade and decompensated cirrhosis. J Hepatol. 2017;66:849–859. 27864004 10.1016/j.jhep.2016.11.001

[26] Burton JR Jr Helmke S Lauriski S Kittelson J Everson GT. The within-individual reproducibility of the disease severity index from the HepQuant SHUNT test of liver function and physiology. Transl Res. 2021;233:5–15. 33400995 10.1016/j.trsl.2020.12.010

[27] Wieland A Etzion O Ali RO Levy E Kleiner DE Helmke SM. HepQuant SHUNT detects portal hypertension in early stages of clinically compensated chronic liver disease. Clin Gastroenterol Hepatol. 2022;20:e890–4. 33895359 10.1016/j.cgh.2021.04.030 PMC8531144

[28] Reiberger T. The value of liver and spleen stiffness for evaluation of portal hypertension in compensated cirrhosis. Hepatol Commun. 2022;6:950–64. 34904404 10.1002/hep4.1855 PMC9035575

[29] Kim HY So YH Kim W Ahn DW Jung YJ Woo H. Non-invasive response prediction in prophylactic carvedilol therapy for cirrhotic patients with esophageal varices. J Hepatol. 2019;70:412–422. 30389550 10.1016/j.jhep.2018.10.018

[30] Ferraioli G Tinelli C Lissandrin R Zicchetti M Bernuzzi S Salvaneschi L. Ultrasound point shear wave elastography assessment of liver and spleen stiffness: Effect of training on repeatability of measurements. Eur Radiol. 2014;24:1283–1289. 24643497 10.1007/s00330-014-3140-y

[31] Stefanescu H Marasco G Calès P Fraquelli M Rosselli M Ganne‐Carriè N. A novel spleen-dedicated stiffness measurement by FibroScan® improves the screening of high-risk oesophageal varices. Liver Int. 2020;40:175–185. 31444849 10.1111/liv.14228

[32] Berger A Shili S Zuberbuhler F Hiriart JB Lannes A Chermak F. Liver stiffness measurement with FibroScan: Use the right probe in the right conditions!. Clin Transl Gastroenterol. 2019;10:e00023. 31009403 10.14309/ctg.0000000000000023 PMC6602786

[33] Brusilovskaya K Königshofer P Lampach D Szodl A Supper P Bauer D. Soluble guanylyl cyclase stimulation and phosphodiesterase-5 inhibition improve portal hypertension and reduce liver fibrosis in bile duct-ligated rats. United European Gastroenterol J. 2020;8:1174–1185. 10.1177/2050640620944140 PMC7724531 32878579

[34] Knorr A Hirth-Dietrich C Alonso-Alija C Härter M Hahn M Keim Y. Nitric oxide-independent activation of soluble guanylate cyclase by BAY 60-2770 in experimental liver fibrosis. Arzneimittelforschung. 2008;58:71–80. 18412020 10.1055/s-0031-1296471

