# Supplementary material for: Safety and pharmacokinetics of BI 685509, a soluble guanylyl cyclase activator, in patients with cirrhosis: A randomized Phase Ib study
Source: Hepatol Commun. 2023 Oct 27;7(11):e0276. doi: 10.1097/HC9.0000000000000276 (PMC10615399; doi:10.1097/HC9.0000000000000276)
Supplement: SUPPLEMENTARY MATERIAL [file hc9-7-e0276-s001.docx]

**Supplemental Digital Content**

**Phase Ib study of the safety and pharmacokinetics of BI** **685509, a soluble guanylyl cyclase activator, in patients with liver cirrhosis**

**First author:** Eric Lawitz^1^

^1^Texas Liver Institute, University of Texas Health, San Antonio, TX, USA

**Supplementary Table 1.** Dose escalation scheme of BI 685509 over 28 days

|  | **BI 685509 dose (mg)** | | | | | | |
| --- | --- | --- | --- | --- | --- | --- | --- |
| **Dose group** | **Day 1**  **(QD)** | **Days 2–3** | **Days 4–9**  **(QD)** | **Days 10–15**  **(QD)** | **Days 16–27**  **(BID)** | **Day 28**  **(QD)** |  |
| Dose group 1 | 0.5 | – | 0.5 | 1.0 | 1.0 | 1.0 |  |
| Dose group 2 | 1.0 | – | 1.0 | 2.0 | 2.0 | 2.0 |  |
| Dose group 3 | 2.0 | – | 2.0 | 3.0 | 3.0 | 3.0 |  |

BID, twice daily; QD, once daily.

**Supplementary Table 2.** Change from baseline to Day 28 in exploratory efficacy endpoints

| **Endpoint** | **CP-A cirrhosis** | | | | **CP-B cirrhosis** | | | |
| --- | --- | --- | --- | --- | --- | --- | --- | --- |
|  | **1 mg BID (n=6)** | **2 mg BID (n=6)** | **3 mg BID (n=6)** | **Placebo (n=6)** | **1 mg BID (n=6)** | **2 mg BID (n=6)** | **3 mg BID (n=7)** | **Placebo (n=6)** |
| **Change from baseline at Day 28, mean (SD)** |  |  |  |  |  |  |  |  |
| Spleen stiffness, kPa | –4.4 (12.3)* | –4.1 (5.4)^†^ | –10.9 (9.0)* | –11.5 (18.6)^‡^ | – | – | – | – |
| Liver stiffness, kPa | 3.0 (8.6) | –0.9 (6.8) | –3.9 (2.8) | –2.9 (12.3) | –9.0 (12.3) | 2.4 (8.5)^§^ | 6.8 (25.3) | –8.9 (13.5) |
| Liver fat content, dB/m | 16.3 (47.0) | –0.5 (69.1) | –36.8 (79.3) | –30.8 (53.2) | 10.7 (41.9) | –9.0 (37.9)^§^ | 0.4 (40.1) | –18.5 (64.2) |
| Portal-systemic shunt fraction, % | 1.0 (7.0) | –5.9 (6.1) | –7.1 (5.3) | –1.2 (7.5)^§^ | – | – | – | – |
| DSI | –0.3 (3.1) | 0.5 (2.1) | –1.8 (1.8) | 0.5 (3.3)^§^ | – | – | – | – |
| STAT, µmol/L | 0.0 (0.3) | –0.3 (0.5) | –0.2 (0.8) | 0.0 (0.5)^§^ | – | – | – | – |
| Pro-C3, µg/L | –1.1 (3.8) | –0.4 (1.7) | 0.9 (1.4) | 0.4 (1.1)^§^ | –0.4 (1.9)^§^ | –0.1 (5.8)^§^ | –2.9 (3.4)^¶^ | –1.7 (4.2)^§^ |
| ELF score | –0.040 (0.242) | –0.015 (0.317) | –0.088 (0.261) | 0.060 (0.254)^§^ | –0.214 (0.293)^§^ | –0.264 (0.754)^§^ | 0.230 (0.603)^¶^ | –0.264 (0.351)^§^ |
| NAFLD fibrosis score | –0.011 (0.398) | –0.060 (0.352) | 0.028 (0.567) | –0.219 (0.677)^§^ | 0.211  (0.330) | 0.184 (0.459)^§^ | 0.193 (0.531)^¶^ | 0.082 (0.418)^§^ |
| Fib-4 | –0.792 (1.273) | –1.791 (2.236) | 0.087 (1.161) | –0.581 (0.908)^§^ | 0.010  (1.114) | –0.581 (1.206)^§^ | 0.175 (0.980)^¶^ | 0.415 (1.994)^§^ |
| APRI | –0.274 (0.405) | –0.525 (0.626) | –0.016 (0.307) | –0.076 (0.073)^§^ | –0.098 (0.237) | –0.244 (0.359)^§^ | 0.042 (0.233)^¶^ | 0.096 (0.474)^§^ |
| MELD | 0.500 (0.548) | 0.167 (1.169) | 0.000 (0.000) | –0.250 (0.957)^‡^ | –0.333 (1.211) | 0.000 (1.225)^§^ | –0.167 (1.472)^¶^ | –0.500 (1.000)^‡^ |
| MELD-Na | 0.887 (1.889) | 0.175 (1.955) | –0.637 (1.443) | –1.062 (2.221)^‡^ | –0.875 (2.157) | –1.695 (3.825)^§^ | 0.512 (2.400)^¶^ | –0.263 (1.533)^‡^ |
| **Absolute values at Day 28, mean (SD)** |  |  |  |  |  |  |  |  |
| Spleen stiffness, kPa | 60.7 (27.1)^‡^ | 55.6 (24.2)* | 51.1 (21.1)^‡^ | 53.6 (15.9)^‡^ | – | – | – | – |
| Liver stiffness, kPa | 32.2 (16.7) | 22.1 (9.0) | 21.2 (3.5) | 25.9 (13.6) | 37.7 (28.6) | 39.2 (24.3)^§^ | 41.1 (25.5) | 19.9 (7.9) |
| Liver fat content, dB/m | 295.7 (40.9) | 295.7 (66.9) | 289.7 (66.7) | 257.3 (56.8) | 284.5 (81.7) | 297.4 (54.2) | 319.0 (70.1) | 268.7 (47.0) |
| Portal-systemic shunt fraction, % | 48.9 (17.3) | 41.0 (12.7) | 42.0 (12.3) | 37.4 (10.8)^§^ | – | – | – | – |
| DSI | 26.1 (7.1) | 26.5 (5.0) | 25.3 (6.0) | 22.2 (5.0)^§^ | – | – | – | – |
| STAT, µmol/L | 1.7 (0.9) | 1.5 (0.8) | 1.7 (1.0) | 1.2 (0.8)^§^ | – | – | – | – |
| Pro-C3, µg/L | 28.1 (12.8) | 16.7 (5.2) | 17.9 (7.0) | 23.3 (15.2)^§^ | 17.5 (1.2)^§^ | 28.2 (16.2)^§^ | 18.6 (7.2)^¶^ | 20.8 (8.3)^§^ |
| ELF score | 11.515 (1.535) | 11.260 (1.075) | 10.787 (1.031) | 10.610 (1.028)^§^ | 11.702 (0.452)^§^ | 11.468 (1.454)^§^ | 10.747 (0.825)^¶^ | 11.464 (0.570)^§^ |
| NAFLD fibrosis score | 1.522 (0.791) | 1.433 (1.452) | 1.853 (2.072) | 1.030 (1.557)^§^ | 2.711  (2.065) | 1.934 (1.088)^§^ | 1.672 (1.835)^¶^ | 3.056 (1.320)^§^ |
| Fib-4 | 4.350 (1.367) | 3.688 (1.802) | 4.687 (2.574) | 3.824 (2.209)^§^ | 5.399  (1.234) | 4.697 (1.359)^§^ | 3.388 (1.411)^¶^ | 7.344 (2.392)^§^ |
| APRI | 0.993 (0.377) | 0.872 (0.479) | 0.960 (0.557) | 0.659 (0.294)^§^ | 0.934  (0.202) | 0.966 (0.407)^§^ | 0.744 (0.364)^¶^ | 1.481 (0.499)^§^ |
| MELD | 8.667 (2.422) | 7.500 (0.548) | 7.667 (1.211) | 7.750 (0.500)^‡^ | 10.167 (2.401) | 9.000 (2.000)^§^ | 8.833 (1.472)^¶^ | 10.750 (2.500)^‡^ |
| MELD-Na | 7.358 (3.419) | 7.217 (2.574) | 7.171 (2.088) | 7.350 (1.900)^‡^ | 9.583  (3.051) | 9.010 (2.037)^§^ | 12.108 (4.228)^¶^ | 12.694 (1.513)^‡^ |

*n=3, ^†^n=2, ^‡^n=4, ^§^n=5, ^¶^n=6

HepQuant parameters (Portal-systemic shunt fraction, DSI, STAT) were assessed at Day 27.

APRI, aspartate aminotransferase to platelet ratio; BID, twice daily; CP-A, Child–Pugh class A; CP-B, Child–Pugh class B; DSI, disease severity index; ELF, enhanced liver fibrosis; Fib-4, fibrosis-4; MELD, model for end-stage liver disease; NAFLD, non-alcoholic fatty liver disease; STAT, d4-cholate at 60 minutes.


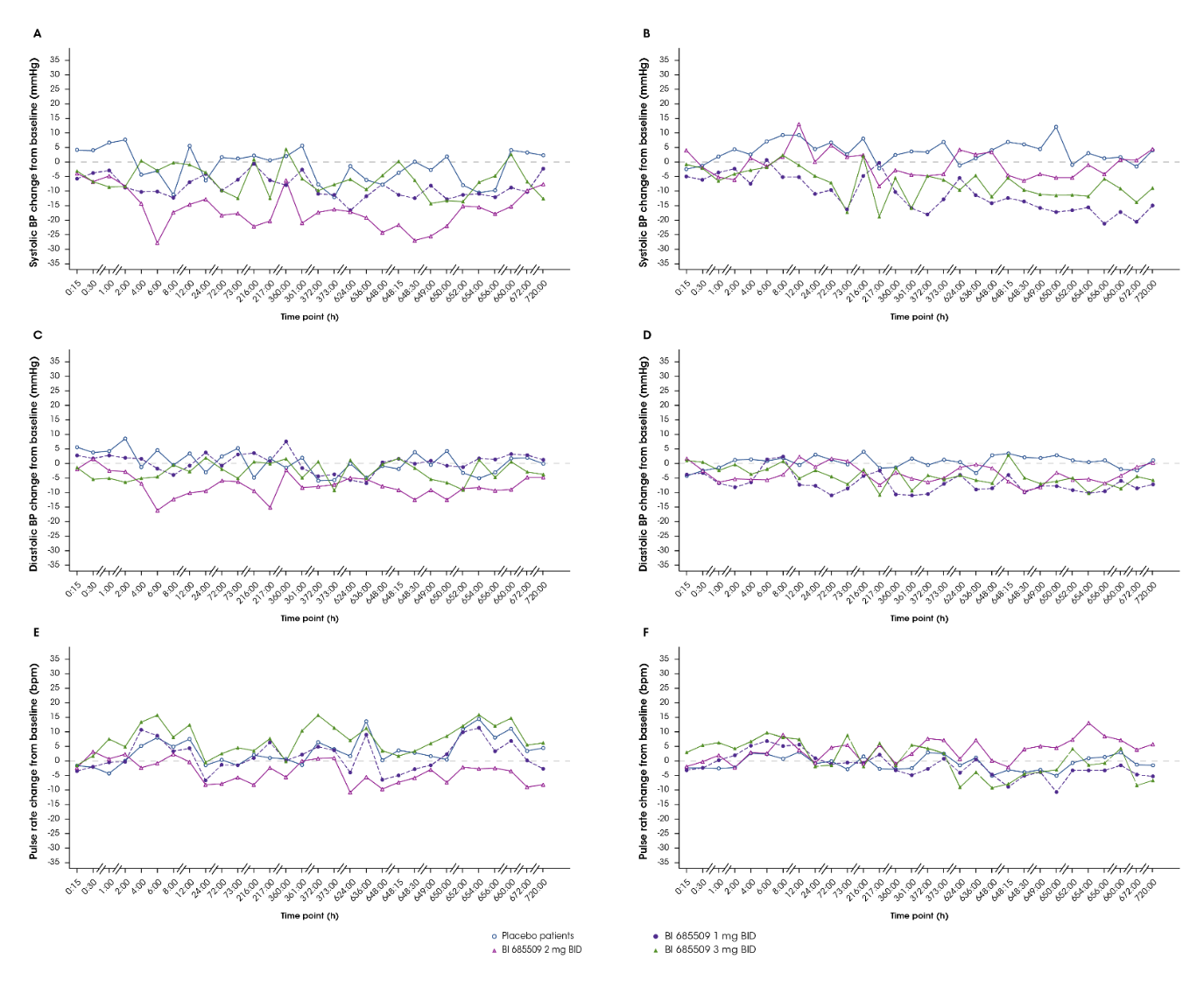
**Supplementary Figure 1.** Change from baseline in vital signs over time following treatment with BI 685509 or placebo. Mean change from baseline in systolic BP in **A**) Child–Pugh class A (CP-A) patients and **B**) CP-B patients. Mean change from baseline in diastolic BP in **C**) CP-A patients and **D**) CP-B patients. Mean change from baseline in heart rate in **E**) CP-A patients and **F**) CP-B patients. Values from early discontinuation are not displayed.

BID, twice daily; BP, blood pressure.

**Supplementary Figure 2.** Mean orthostatic change from supine to standing in systolic BP and pulse rate over time. Mean orthostatic change in Child–Pugh class A patients treated with 1 mg twice daily BI 685509 for **A**) systolic BP and **B**) pulse rate. Mean orthostatic change in Child–Pugh class B patients treated with 3 mg twice daily BI 685509 for **C**) systolic BP and **D**) pulse rate.


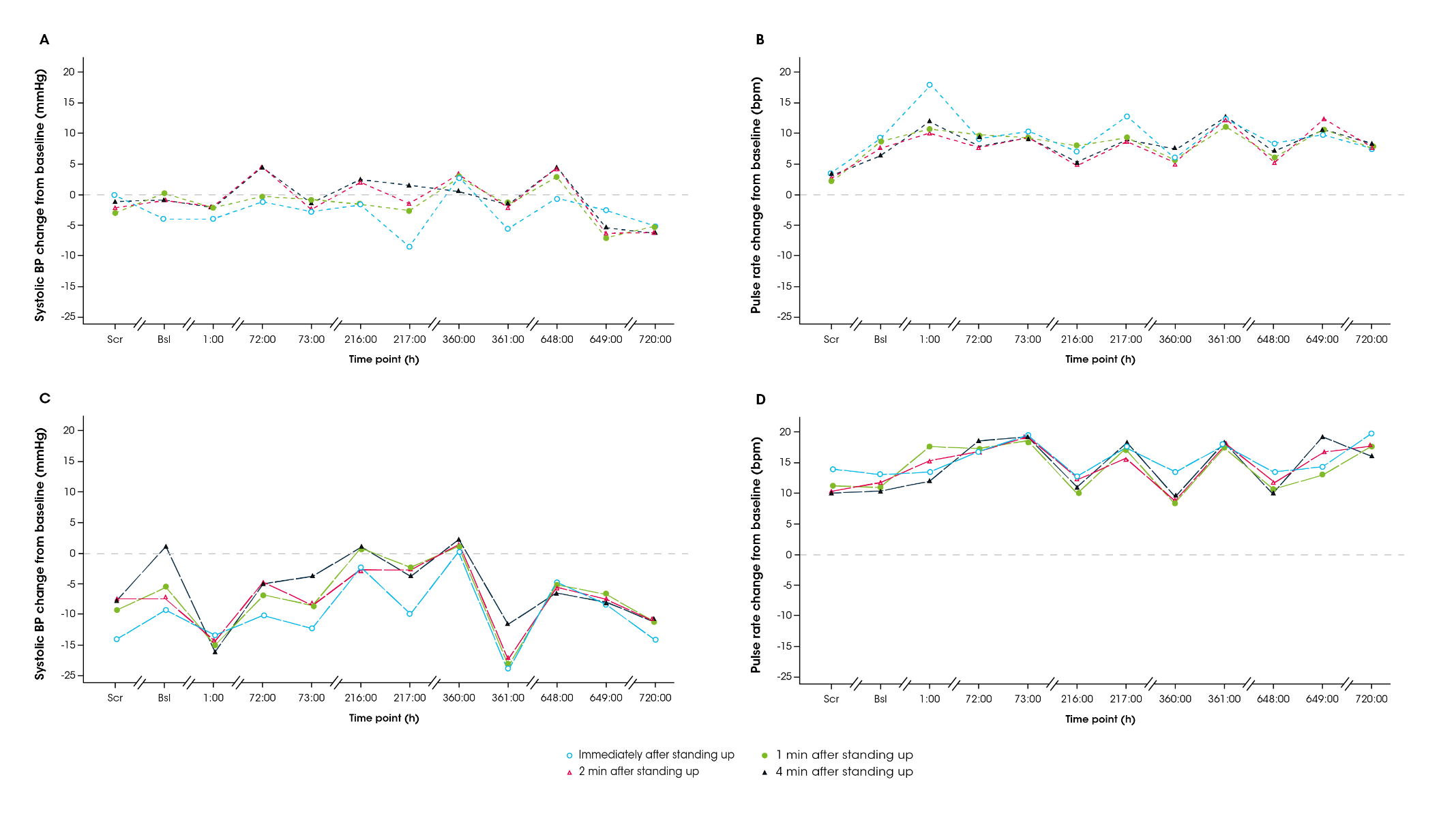


BP, blood pressure; Bsl, baseline; Scr, screening.
